# *In Vitro* Evaluation of Antibacterial Potential of Dry FruitExtracts of Elettaria cardamomum Maton (Chhoti Elaichi)

**Published:** 2010

**Authors:** Purshotam Kaushik, Pankaj Goyal, Abhishek Chauhan, Garima Chauhan

**Affiliations:** *Department of Botany and Microbiology, Gurukul Kangri University, Hardwar, Uttarakhand, India.*

**Keywords:** Antibacterial activity, *Elettaria cardamomum*, Chhoti elaichi, Minimum inhibitory concentration

## Abstract

Emergence of resistance among pathogenic bacteria against available antibiotics is posing a great challenge to the current world. Thus, there is a great need to discover novel antibiotics. Traditional plants have been proved to be novel source in the search of antimicrobial compounds. The current study pertained to the susceptibilities of some clinically significant bacterial species to various crude extracts of *Elettaria cardamomum *Maton (Chhoti elaichi) dry fruits by agar well diffusion assay. Minimum inhibitory concentrations (MIC) of extracts were further evaluated against these bacteria. The study indicated that antibacterial activity of this plant is dependent on the type of extract and the organism evaluated. Ethanol extract was found to have comparatively higher activity than other organic and aqueous extracts. Gram-positive bacteria showed competent but variable susceptibilities to all the tested extracts. MIC data showed hopeful results as some of the extracts exhibited significant inhibitions of bacteria even at concentrations as low as 512 μg/mL. Overall, *E. cardamomum *seems to have significant antibacterial activity and to be very useful in the discovery of novel antibiotic.

## Introduction

Antibiotic chemotherapy has been one of the most important medical achievements of the twentieth century. This therapy is widely practiced for the treatment of various microbiological infections. In recent years, the prevalence of antimicrobial resistance among key microbial pathogens such as *Staphylococcus aureus*, *Streptococcus pneumoniae*, *Klebsiella*, *Haemophilus*, *Neisseria*, *Moraxella *and *Enterococcus faecalis *is increasing at an alarming rate worldwide (1-3). The outcome is that many antibiotics can no longer be used for the treatment of infections caused by such organisms and the threat to the usage of other drugs is steadily increasing (4, 5). A feasible way to combat the problem of microbial resistance is the development of new antibacterial agents for substitution with ineffective ones. Natural products particularly plant-based products have played an important role throughout the world in treating and preventing human diseases (6-8). Thus, they may become the base for the development of a medicine, a natural blueprint for the development of new drugs (9). Furthermore, many herbs used by Ayurvedic practitioners show promising results and could be appropriate for larger randomized trials. It is presumed that the broad-spectrum effectiveness of these spices may provide a suitable basis for new antimicrobial therapies (10).

*Elettaria cardamomum *Maton is an important member of family Zingiberaceae. Small cardamoms are popularly known as ‘chhoti elaichi’ or the ‘true cardamom’ or ‘*Ela*’. Chhoti elaichi has been the second most important ‘National Spice” of India and is also rightly known as the ‘Queen of Spices’ (11). This leafy perennial herb is originated from India and Sri Lanka and is commonly cultivated in southern India. Fruits and seeds are economically important parts of the plant. The seeds contain essential oil in concentration of about 4% of dry weight. The main compound is 1,8-cineole (representing 50% or more), with smaller amounts of α-terpineol, borneol, camphor, limonene, α-terpenyl acetate, and α-pinene (12, 13). Indian cardamom is low in fat and high in protein, iron, and vitamins B and C. Cardamom seeds, with their sweet and spicy aroma, are used in aromatherapy to stimulate energy (14, 15). It also acts as Ayurvedic aphrodisiac and remedy in case of digestive problems, asthma, bronchitis, and urinary complaints and several other human ailments (16, 17).

The present study was aimed to evaluate the antibacterial potential of dry fruits extracts of *E. cardamomum *against some selected bacterial species.

## Experimental

*Plant material*

*Elettaria cardamomum *dry fruits were collected locally in late October of 2007. The plant was taken to the laboratory and was authenticated by Professor P. Kaushik at Department of Microbiology, Gurukul Kangri University, Hardwar (India). 

*Extract preparation*

Dry fruits of *E. cardamomum *were extensively washed under running tap water for removal of dust particles and epiphytic hosts normally found on the surface, followed by washing with sterilized distilled water. They were further air-dried on filter paper at room temperature and then powdered with the help of sterilized pestle and mortar under aseptic condition. Dry powder was further extracted by using aqueous and organic solvents (18, 19) as follows:

*Aqueous extraction*

Air-dried powder (10 g) of the respective plant part was mixed well in 100 mL sterilized distilled water and kept at room temperature for 24 h on an orbital shaker with 150 rpm. The solution was further filtered using muslin cloth. The filtrate was centrifuged at 5000 rpm for 15 min. The supernatant thus obtained was filtered through Whattman filter No. 1 under strict aseptic conditions and the filtrate was collected in a preweighed sterilized test tube. Aqueous extracts were prepared in final concentration of 100 mg/mL. Test tubes were cotton plugged and stored in refrigerator until further used.

*Organic solvent extraction*

Air-dried powder (10 g) of the respective plant part was thoroughly mixed with 100 mL organic solvent (ethanol, methanol, ethyl acetate and hexane). The mixture was placed at room temperature for 24 h on orbital shaker at 150 rpm. Solution was filtered through muslin cloth and then re-filtered by passing through Whattman filter No. 1. The filtrate thus obtained was concentrated by complete evaporation of solvent at room temperature to yield the pure extract. Stock solutions of crude extracts from each type of organic solvent were prepared by mixing well the appropriate amount of dried extract with the respective solvent to obtain a final concentration of 100 mg/mL. Each solution was stored in refrigerator after collecting in sterilized bottles until further used.

*Bacterial strains*

A total of six bacterial strains including both Gram-negative and Gram-positive bacteria (*Escherichia coli *MTCC-739, *Salmonella typhi *MTCC-531, *Bacillus cereus *MTCC-430, *Bacillus subtilis *MTCC-736, *Streptococcus pyogenes *MTCC-442, and *Staphylococcus aureus *MTCC-740) were selected to assess susceptibility patterns against the extracts prepared in the present study. All these bacterial species are recommended by ATCC for their susceptibility assay. The bacterial cultures were maintained on nutrient agar slants at 5°C in refrigerator. Each of the microorganisms was reactivated prior to susceptibility testing by transferring them into a separate test tube containing nutrient broth and incubated overnight at 37°C.

*Antibacterial susceptibility assay*

Extracts obtained by various processes were evaluated for their potential antibacterial activities by the standard agar well diffusion assay (20). All extracts were sterilized by sterile membrane syringe filter (pore size 0.45 μm, manufactured by Pall Life Sciences). Petri dishes (90 mm) containing 18 mL of Mueller-Hinton Agar were seeded with approximately 100 μL inoculum of bacterial strain (inoculum size was adjusted so as to deliver a final inoculum of approximately 10^8^ colony forming units (CFU/mL). Media was allowed to solidify. Wells of 6 mm diameter were cut into solidified agar media using a sterilized cup-borer. 100 μL of each extract was poured in the respective well and the plates were incubated at 37°C overnight. The antibacterial activity of each extract was expressed in terms of the mean of diameter of zone of inhibition (in mm) produced by each extract at the end of incubation period.

Sterilized distilled water and other solvents used in preparation of extracts were used as negative control. Tetracycline (5 μg/mL) was used as a standard antibiotic (i.e. positive control) in the present study for a comparative analysis with the effectiveness of various plant extracts against selected bacterial species.

*Assessment of minimum inhibitory concentration*

Active extracts obtained by agar well diffusion assay were further subjected to determine the MIC required for the bacteriostatic effects by standard two-fold broth microdilution methodology (21). A stock solution of each active extract was serially diluted in 96-well microtiter plate with Mueller-Hinton broth to obtain a concentration ranging from 8.0 μg/mL to 4096 μg/mL. A standardized inoculum for each bacterial strain was prepared so as to give an inoculum size of approximately 5×10^5^ CFU/mL in each well. Microtiter plates were then kept at 37°C for an overnight incubation. Following incubation, the MIC was calculated as the lowest concentration of the extract inhibiting the visible growth of bacterial strain using reflective viewer.

All the chemical ingredients used in present study were of analytical grade and were purchased from Hi Media, India.

*Phytochemical analysis*

Active crude extract of the fruits was further analyzed for phytochemical constituents by using standard methods (22, 23).

*Statistical analysis*

Agar well diffusion assay was performed in triplicate under strict aseptic conditions to ensure consistency of all findings. Data of all experiments were statistically analyzed and expressed as mean ± standard deviation (SD).

## Results and Discussion

Antibacterial activity of different extracts prepared from dry fruits of *E. cardamomum *(small cardamom) was expressed in terms of zone of inhibitions (Table 1). The pattern of inhibition varied with the type of solvent used for extraction and the microorganism tested for susceptibility assay. Data are the indicative of almost equal activity of aqueous and ethyl acetate extracts. Aqueous extract was found inhibitory for all the test bacteria and *S. aureus *was found the most susceptible bacterium to the extract. Ethyl acetate extract was active against all microflora except *S. typhi*. Ethanol extract was found inhibitory against all the test bacteria but the activity was found lower than previously discussed two extracts. Methanol extract was active only against *S. aureus *and *E. coli*. Hexane extract was found completely inactive against all the test organisms. Inhibition range for *S. typhi *and *S. pyogenes *was observed very mild against ethanol and aqueous extracts.

**Table 1:** *In-vitro *antibacterial activity of aqueous and organic extracts of *Elettaria cardamomum *dry fruits.

**Sample**	Diameter of zone of inhibition (mm)*					
	**Gram-negative bacteria**		**Gram-positive bacteria**			
	***E. coli***	***S. typhi***	***S. pyogenes***	***S. aureus***	***B. subtilis***	***B. cereus***
Ethanol extract	10.77 ± 0.92	09.67 ± 0.76	09.06 ± 0.51	14.80 ± 0.72	10.53 ± 0.50	09.87 ± 0.81
Methanol extract	11.46 ± 0.81	NI	NI	15.80 ± 2.03	NI	NI
Ethyl acetate extract	13.00 ± 0.87	NI	11.63 ± 0.55	19.80 ± 1.71	11.5 ± 0.87	10.83 ± 1.76
Hexane extract	NI	NI	NI	NI	NI	NI
Aqueous e xtract	14.67 ± 0.29	12.27 ± 1.10	10.88 ± 0.83	20.63 ± 0.71	11.55 ± 0.51	11.22 ± 1.07
Tetracycline***	29.50 ± 0.50	25.83 ± 1.61	29.83 ± 1.89	32.50 ± 1.50	34.17 ± 1.76	32.16 ± 1.04
Ethanol	NI	NI	NI	NI	NI	NI
Methanol	NI	NI	NI	NI	NI	NI
Ethyl acetate	NI	NI	NI	NI	NI	NI
Hexane	NI	NI	NI	NI	NI	NI

*Values of the observed diameter zone of inhibition (mm) including the diameter of well (6 mm) after 24 h incubation against different bacterial species when subjected to different extracts in agar well diffusion assay. In each well, the sample size was 100 μL. Inhibition observed in extracts due to solvent were assessed through negative controls.

**NI: no inhibition zone was observed.

*** Tetracycline (5 μg/mL) was used as standard antibiotic.

Table 2 represents the MIC values of various active crude extracts of *E. cardamom *against susceptible bacteria. All the tested extracts showed significant variations in MIC values depending upon the test bacteria. *S. aureus*, the most sensitive bacteria showed the variable MIC ranges. MIC was not observed against *S. typhi *in case of aqueous extract and against *E. coli *in case of ethyl acetate extract. Aqueous extract could inhibit *E. coli *at moderate concentrations. All the extracts showed comparatively higher MIC values than that of tetracycline (a standard antibiotic used in present study). 

**Table 2:** Minimum inhibitory concentration of active crude extracts of *Elettaria cardamomum *dry fruits

**Type of active ** **crude extract**	**Test microorganism**	Concentration of extracts (μg/mL)*										MIC (μg/mL)
		**4096**	**2048**	**1024**	**512**	**256**	**128**	**64**	**32**	**16**	8	
Ethanol	*S. aureus*	-	+	+	+	+	+	+	+	+	+	4096
Methanol	*S. aureus*	-	-	+	+	+	+	+	+	+	+	2048
Ethyl Acetate	*S. aureus*	-	-	-	+	+	+	+	+	+	+	1024
Ethyl Acetate	*E. coli*	+	+	+	+	+	+	+	+	+	+	> 4096
Aqueous	*E. coli*	-	-	+	+	+	+	+	+	+	+	2048
Aqueous	*S. typhi*	+	+	+	+	+	+	+	+	+	+	> 4096
Aqueous	*S. aureus*	-	-	-	-	+	+	+	+	+	+	512
Tetracycline	*S. aureus*	-	-	-	-	-	-	-	-	-	-	< 8

*Different concentrations of active crude extracts evaluated in 96-well microtiter plate using microbroth dilution assay as recommended by NCCLS. All values are expressed in μg/mL.

The (-) represents ‘no growth observed, and the (+) represents ‘growth observed’.

Phytochemical analysis revealed the presence of alkaloids, tannins, terpenoids and flavonoids in aqueous extract of the studied plant material. 

The results of our study are in agreement with the study of Arora and Kaur (24), who reported that the water extracts of fruits of *E. cardamomum *(small cardamom) were effective against several human pathogenic bacteria with zone sizes ranging from 15 to 28 mm. However, their results were in contrast to the study of Ahmad *et al. *(25) who found no antibacterial activity using aqueous extracts. The reason given for the variations observed was method of extraction or strain differences. Nanasombat and Lohasuthawee (26) found the activity of ethanol extracts of fruits in the range of 7 mm to 12 mm against almost all the strains tested. Agaoglu et al. (12) evaluated the diethyl ether extract of cardamom seed against clinically significant microorganisms. The extract was found most inhibitory against *S. aureus*, the least inhibition was found against *E. coli*. Higher susceptibilities of Gram-positive bacteria may be attributed probably due to the differences in chemical composition and structure of cell wall of both types of microorganisms. 

Solvents including methanol, ethanol, ethyl acetate and hexane were used in the current study to prepare crude extracts. These solvents were further evaluated separately as negative control for their antibacterial activity to check whether the activity is due to the extracts containing the active compound(s) or due to the solvents used for the extraction. Our data (Table 1) indicated that zone of inhibition arises due to the extract, as there was no any inhibition zones appeared against these solvents. Considering the fact that these solvents are toxic, there must be higher zone of inhibitions or there must be presence of zone of inhibition in every case of extract as they are prepared by any of these solvents (27).

Our findings support the traditional medicinal use of this plant and its future aspects in developing novel antimicrobials. *Elettaria cardamomum *can potentially be used in the treatment of various infectious diseases caused by microorganisms that are showing resistance to currently available antibiotics. Furthermore, active plant extracts can be subjected to various chemical evaluations by several methods such as GC-MS, NMR (nuclear magnetic resonance), Mass Spectrometry, etc. for the isolation of the therapeutic antimicrobials.
